# Multiple displacement amplification as an adjunct to PCR-based detection of *Staphylococcus aureus *in synovial fluid

**DOI:** 10.1186/1756-0500-3-259

**Published:** 2010-10-13

**Authors:** Sandeep Kathju, Roger S Lasken, Latha Satish, Sandra Johnson, Paul Stoodley, J Christopher Post, Garth D Ehrlich

**Affiliations:** 1 Center for Genomic Sciences, Allegheny-Singer Research Institute, Allegheny General Hospital, Pittsburgh, PA, USA; 2 Division of Oral/Maxillofacial Surgery, Allegheny General Hospital, Pittsburgh, PA, USA; 3 Division of Otolaryngology/Head and Neck Surgery, Allegheny General Hospital, Pittsburgh, PA, USA; 4 Division of Plastic Surgery, Allegheny General Hospital, Pittsburgh, PA, USA; 5 Department of Microbiology and Immunology, Drexel University College of Medicine, Allegheny Campus, Pittsburgh, PA, USA; 6 National Center for Advanced Tribology at Southampton (nCATS), University of Southampton, Highfield, Southampton, SO17 1BJ, UK

## Abstract

**Background:**

Detection of bacterial nucleic acids in synovial fluid following total joint arthroplasty with suspected infection can be difficult; among other technical challenges, inhibitors in the specimens require extensive sample preparation and can diminish assay sensitivity even using polymerase chain reaction (PCR)-based methods. To address this problem a simple protocol for prior use of multiple displacement amplification (MDA) as an adjunct to PCR was established and tested on both purified *S. aureus *DNA as well as on clinical samples known to contain *S. aureus *nucleic acids.

**Findings:**

A single round of MDA on purified nucleic acids resulted in a > 300 thousand-fold increase in template DNA on subsequent quantitative PCR (qPCR) analysis. MDA use on clinical samples resulted in at least a 100-fold increase in sensitivity on subsequent qPCR and required no sample preparation other than a simple alkali/heat lysis step. Mixed samples of *S. aureus *DNA with a 10^3 ^- 10^4^-fold excess of human genomic DNA still allowed for MDA amplification of the minor bacterial component to the threshold of detectability.

**Conclusion:**

MDA is a promising technique that may serve to significantly enhance the sensitivity of molecular assays in cases of suspected joint infection while simultaneously reducing the specimen handling required.

## Background

Although molecular detection of pathogens in clinical samples by PCR has been widely investigated, its reliability is still questioned in some clinical scenarios. For suspected infection following total joint arthroplasty, PCR of 16S rDNA remains controversial as evidence for pathogen presence and ongoing infection. Some reports consider PCR-based techniques overly sensitive, potentially indicating DNA even in the absence of clinical criteria for infection [1]. Alternatively, it has been posited that so-called "aseptic" loosening of prostheses may be due to low-grade infection that remains difficult to detect even with molecular techniques [2], and that false-negatives may result from inefficient DNA extraction from target organisms (especially Gram-positives), loss of DNA during sample processing, or the presence of PCR inhibitors in clinical samples [3].

In synovial fluid the problem of PCR inhibitors is significant, and virtually all studies evaluating orthopedic joint infections with PCR techniques have first undertaken some means of isolating genomic DNA from the clinical specimens [eg. [4-6]]. van der Heijden et al., for example, employed a lysis buffer containing SDS and proteinase K at 60C for 18 hours, followed by phenol extraction and ethanol precipitation, but found that additional purification with a QIAamp blood kit (Qiagen) was still necessary [7]. As a result of these various concerns, PCR-based analysis of clinical joint aspirates is not typically used as an aide to clinical decision-making when confronted with a possibly infected joint prosthesis.

A growing body of evidence supports the theory that chronic prosthetic joint infections arise from bacterial biofilms associated with the implants. Biofilm bacteria are notoriously difficult to culture and highly resistant to conventional antibiotic therapy. Treatment of the infected implant often requires explantation, inflicting significant morbidity and entailing significant expense; surgeons are understandably reluctant to undertake such a course without good reason, but standard bacterial culture of diagnostic aspirates is usually negative. When culture results are positive, *S. aureus *is the most frequently recovered organism in chronic joint implant infections, although numerous unusual bacterial species have also been implicated [8]. It would be an important advance to establish a diagnostic protocol for such infections that reliably improved sensitivity with a minimum of specimen handling.

We have used multiple displacement amplification (MDA) [9,10] to improve PCR detection of bacterial DNA present in clinical samples using *S. aureus *as a test organism. MDA amplifies all DNA content in a sample including human and bacterial DNA [11,12]. It facilitates subsequent PCR detection of bacteria by 1) enriching DNA relative to PCR inhibitors (such as hemoglobin and nucleases), 2) generating abundant DNA template with less incidence of mutational error than PCR amplification where low bacterial counts provide only suboptimal amounts of DNA, and 3) providing a large source of DNA to carry out replicate PCR assays, repeat assays to confirm results, interrogate an unlimited number of genomic loci and any pathogen within a mixed sample, and archive DNA for genomic sequencing or other detailed studies. MDA is simple to employ and requires no specialized equipment. Thus it may be extremely useful in investigating complex clinical samples containing comparatively small quantities of target organisms.

## Methods

### Quantitative PCR assay for *S. aureus *gene loci

In order to quantify the effect MDA can have, we first established and validated a quantitative real-time PCR assay for several single copy (not rDNA) gene loci in *S. aureus*: lysS and proS. The primers and probes used are listed in Table 1. 50 μl PCR reactions (containing varying concentrations of *S. aureus *genomic DNA) used denaturation at 95C × 10 min; then 2-stage PCR at 95C × 15 sec, and 60C × 1 min × 40 cycles total, with 0.5 μl Platinum Taq polymerase (Invitrogen), 1× PCR buffer, 5 mM MgCl_2_, dNTPs at 1 mM, primers and Taqman probe at 500 nM, and 1 μl of Rox reference dye. Assays were performed in an ABI 7900HT Sequence Detection System and critical cycle values were determined with the manufacturer's software.

**Table 1:** Primer and probe sequences used in qPCR assays for lysS and proS

lysS primers	5'-GTCGTTTTGAAGCGCAACTT-3'
	5'-CCTGTCGGAGGCATACCATA-3'

lysS probe	5'-GCGCAAGGTAATGATGAAGCGCA-3' (6-FAM)

proS primers	5'-GGCATTAAGTGCTATCGGTGA-3'
	5'-AAAGGTTGCACAGTAGAATGCTT-3'

proS probe	5'-GAAAAAGCAGAAGTCGTTTACGAACC-3' (6-FAM)

### Quantitation of purified *S. aureus *DNA after pre-treatment with MDA

We first investigated the effect of MDA on purified genomic DNA using the GenomiPhi MDA kit (GE-Healthcare). Varying amounts of *S. aureus *DNA (isolated as per [13] from reference strain Seattle 1945, ATCC# 25923) from 0.01 ng to 100 ng were assayed using the qPCR assays described above and a standard curve was established. 0.01 ng of genomic *S. aureus *DNA were also pre-incubated with 1 μl of the GenomiPhi enzyme for 16 hours at 30 C in 100 μl volume (as per the manufacturer's specifications). A corresponding control reaction lacking only the GenomiPhi enzyme itself was also carried out. 5 μl of the resulting material (effectively a 1/20 dilution) were then assayed for DNA content by qPCR as described above.

### Assay for *S. aureus *DNA in clinical samples using MDA

MDA was next tested for *S.aureus *DNA in synovial fluid from an elbow total joint arthroplasty patient where infection was suspected but could not be confirmed by standard microbiology despite two years of recurrent symptoms and multiple surgical and medical interventions. We previously reported that bacteria in biofilm configuration were observed in aspirate, tissue, and cement samples from this patient by confocal microscopy, and that PCR and reverse-transcriptase PCR detected intact *S. aureus *(but not *S. epidermidis*) DNA and mRNA respectively in purified nucleic acids from the synovial fluid aspirate [14]. Two discrete samples were now subjected to qPCR analysis with and without pre-treatment with MDA.

Elbow synovial fluid aspirate obtained with sterile technique was placed either in RNAlater solution or in a microfuge tube directly, then snap frozen in dry/ice ethanol and preserved at -70C until further processing. On thawing, 0.1 μl, 1 μl, or 10 μl of stored aspirate was brought to a volume of 27 μl with phosphate buffered saline (PBS), then lysed by addition of 1/10 vol (3 μl) of 400 mM KOH, 10 M EDTA with incubation at 65C × 3 minutes. The lysates were then neutralized by addition of 3 μl of 800 mM Tris-HCl (~ pH 4), for a total volume of 33 μl. As controls, the same volumes of stored aspirate (0.1, 1 and 10 μl) were brought directly to 33 μl with added PBS and were not lysed. Samples to undergo MDA had 67 μl of MDA mix added, comprised of 25 μl 4× MDA master mix, 41 μl water, and 1 μl of Phi 29 enzyme. Control samples that were not to undergo MDA were prepared similarly, except with 42 μl of water and no GenomiPhi DNA polymerase. All samples were incubated at 30C × 16 hours, then at 65C × 3 minutes (to deactivate the DNA polymerase) before being stored at 4C. 20 μl aliquots were then used in qPCR as described above (biological duplicates of each assay condition gave identical results).

### Quantitation of *S. aureus *DNA and human genomic DNA in aspirate samples

Total nucleic acids were isolated from two discrete samples of elbow aspirate fluid as per Stoodley et al. [14]. This material was then used as substrate for direct quantitation of *S. aureus *DNA by real-time PCR with the proS and lysS assays described above and in Table 1. The quantity of human genomic DNA contained was determined by quantitative PCR for human GAPDH using Gene Expression Assays from Applied Biosystems (Foster City, CA); assay numbers are Hs02786624-g1 and Hs03929097-g1.

### Assay for *S. aureus *DNA in samples of mixed human and bacterial DNA

0.01 ng of purified *S. aureus *DNA was mixed with increasing amounts (0 ng to 100 ng) of purified human genomic DNA. These mixes underwent MDA using the GenomiPhi V2 DNA Amplification kit (GE Healthcare, cat. # 25-6600-30) as per the manufacturer's instructions. Briefly, mixes containing 0.01 ng of *S. aureus *DNA with varying quantities of human DNA were brought to 10 μl in Sample Buffer, then heat denatured at 95C × 3 min, then placed on ice. 10 μl of Master Mix (including reaction buffer and enzyme) were added to each sample and allowed to incubate at 30C × 1.5 hrs. The enzyme was then inactivated at 65C × 10 min, then cooled to 4C. Control reactions lacking only the GenomiPhi enzyme were also run. 1 μl of the final reaction products were then diluted 1:100 in TE, and these diluted products were then used in qPCR assays for *S. aureus *with the lysS and proS loci as described above.

## Results

We first established a qPCR assay for *S. aureus *based on the single copy loci lysS and proS. qPCR of both target genes yielded amplimers of the expected molecular weight on gel electrophoresis (data not shown) and showed a direct correlation between critical cycle threshold and substrate concentration. Appropriate sigmoidal curves for amplimer accumulation were observed in all instances.

We next determined what degree of amplification might be afforded by MDA on the naked DNA template used to validate our *S. aureus*-specific assays. Pre-incubation with the GenomiPhi enzyme resulted in a vast increase in the amount of target *S. aureus *DNA present, reducing the threshold critical cycle from ~ 31.7 to ~16.9. This represents a theoretical amplification (assuming a two-fold exponential increase with each cycle reduced, and allowing for the 20-fold dilution introduced for experimental purposes) of > 2^14 ^× 20, or over 300 thousand-fold (Table 2).

**Table 2:** Quantitation of *S. aureus *genomic DNA (gDNA) by real time PCR

MDA	S. aur gDNA	Probe	Average Ct	SEM (±)
N	100 ng	proS	17.8	0.03
N	10 ng	proS	22.1	0.05
N	1 ng	proS	24.6	0.03
N	0.1 ng	proS	27.7	0.06
N	0.01 ng	proS	31.7	0.26
N	0 ng	proS	U	U
(N)	0.01 ng	proS	35.4	0.85
(Y)	0.01 ng	proS	16.9	0.05

Triplicate samples of isolated *S. aureus *genomic DNA were assayed by qPCR; the calculated average threshold critical cycle (Ct) values are provided, as is the standard error. Each sample concentration gave a highly reproducible result; the data shown are using the proS probe, but equivalent results were obtained using lysS. Pre-treatment of *S. aureus *gDNA by MDA resulted in a massive reduction in Ct, indicating a massive amplification of template. In MDA control lacking the DNA polyermase, Ct actually slightly increased, due to dilution of the starting material. 0 ng of gDNA (negative control) gave no detectable signal (Ct undetermined, represented as "U" above).

We next examined what effect MDA pre-treatment would have on clinical samples of joint aspirates recovered from a well-characterized infected patient after total elbow arthroplasty; both samples stored in RNAlater and also snap-frozen directly were tested. In order to make available DNA for amplification without need for extensive specimen handling, a simple protocol for alkali/heat lysis was introduced. Results are shown in Table 3.

**Table 3:** qPCR assay of aspirated synovial fluid, +/- lysis and MDA

**Sample vol**.	Probe	Lysis -, MDA -	Lysis +, MDA -	Lysis +, MDA +
0.1 μl	proS	U	+	+
1.0 μl	proS	U	U	+
10 μl	proS	U	U	+
0.1 μl	proS	U	+	+
1.0 μl	proS	U	U	+
10 μl	proS	U	U	+
0.1 μl	lysS	U	+	+
1.0 μl	lysS	U	U	+
10 μl	lysS	U	U	+
0.1 μl	lysS	U	+	+
1.0 μl	lysS	U	U	+
10 μl	lysS	U	U	+

Varying volumes of joint aspirate were treated as per the conditions listed atop each column, either with or without lysis and MDA. "U" indicates that no critical cycle value was obtained (undetermined), and thus that no template DNA was detected. "+" indicates that a valid Ct value was obtained, and an appropriate sigmoidal accumulation curve, indicating successful detection of template. Results were identical in two samples of aspirate, using the proS and lysS probes.

When aspirate samples were unlysed there was no signal accumulation above the detection threshold, consistent with the presence of inhibitors since these same materials have yielded PCR amplimers successfully after purification of nucleic acids within. Aspirate stored in RNA later as well as stored directly, when lysed, did give a positive signal on direct qPCR assay but only when 0.1 μl of material was used, not when greater quantities of starting material were present, again consistent with the presence of inhibitors. In contrast, all lysed aspirates subjected to MDA gave convincingly positive qPCR signals for both proS and lysS, demonstrating that pre-amplification with MDA expands the window of detectability in these joint aspirate samples by at least 100-fold (from 0.1 μl to 10 μl inclusive). Use of RNAlater as a storage medium had no effect on the initial detection or ultimate amplification yield.

We next used real-time PCR to quantify the amount of *S. aureus *and human genomic DNA present in synovial fluid samples. Assaying two independently obtained samples from the same patient, we found that *S. aureus *DNA was present at 0.014 μg/ml in one sample, and at 0.020 μg/ml in the other. Human genomic DNA was present in over a thousand-fold excess by comparison, measured at 33.6 μg/ml and 79.4 μg/ml respectively.

In order to verify that MDA could function to amplify minor quantities of bacterial DNA in the presence of large (but physiologically relevant) quantities of human DNA, we next mixed small quantities of purified *S. aureus *DNA (in roughly the quantity we would expect to find in a small volume of synovial fluid) with increasing amounts of purified human genomic DNA. Samples were either treated +/- MDA; the resulting mixes were diluted and then assayed by quantitative PCR. Results are shown in Table 4.

**Table 4:** qPCR assay of purified *S. aureus *and human DNA (hDNA) mix, with and without MDA pre-treatment

Sample	Probe	MDA -	MDA +
0.01 ng S. aur + 100 ng hDNA	proS	U	+* (10/12)
0.01 ng S. aur + 10 ng hDNA	proS	U	+ (12/12)
0.01 ng S. aur + 1 ng hDNA	proS	U	+ (12/12)
0.01 ng S. aur + 0 ng hDNA	proS	U	+ (12/12)

Ct values were undetermined (U) for *S. aureus *DNA without MDA pre-treatment. In contrast, "+" indicates the successful detection of target template, demonstrating that MDA had successfully increased the *S. aureus *component to the level of detection; the numbers at right in parentheses represent the number of successful amplification attempts/number of total attempts. When 100 ng of human genomic DNA were used, in 2 out of 12 attempts no signal detection was observed although results were positive for the majority of trials, thus prompting a "+*" notation, with the possibility that this amount of excess background DNA may become partly inhibitory to the MDA.

Using 0.01 ng of *S. aureus *DNA as a starting template, we were unable to detect any accumulation signal in our real-time assay under any condition when pre-treatment with MDA had not been applied. In contrast, when pre-treatment with MDA was effected, all samples gave convincing positive results, despite the additional dilution. Results were uniformly positive when up to 10 ng of human DNA were in the initial mix, representing a thousand-fold excess. Results were still mostly positive, in 10 out of 12 trials, when 100 ng of human DNA were in the initial template mix, raising the possibility that a > 10^4^-fold excess of human DNA may itself begin to exert some inhibitory effect on the ability of MDA to amplify minor bacterial components in the mix. However, in general these results support our direct evidence from the clinical samples that MDA has the potential to be a useful adjunct to PCR examination of synovial aspirates with its ability to amplify small quantities of bacterial DNA in complex mixes.

## Discussion

The technique of multiple displacement amplification relies on the unique properties of the Phi29 DNA polymerase, which generates copies of its template DNA with great fidelity; the calculated error rate of the Phi29 polymerase is 3 × 10^-6^, almost two orders of magnitude superior to that of Taq polymerase. The Phi29 catalyzed reaction proceeds isothermally through a mechanism of strand displacement, and the enzyme is highly processive, typically incorporating > 70,000 bp per binding event [15]. These features, markedly different from those of PCR-based amplification, make MDA a potentially useful adjunct to quantitative PCR assays without being subject to the same technical pitfalls as PCR.

MDA has been used for a variety of purposes from a number of sample types. MDA improved sensitivity of PCR detection of rare *Wolbachia *cells in mites by 10^2^-10^7^-fold [16]. Genomic DNA was amplified by MDA from lepromatous human tissue, improving the success of microsatellite analysis of *Mycobacterium leprae *from 20% to 92% for these highly degraded archived samples [17]. It has been successfully used to amplify whole genome DNA from a wide variety of clinical specimens [11] and from very limited numbers of cells or even single cells [18-20].

This report represents (to our knowledge) the first application of MDA to diagnostic challenges within the orthopedic realm. Although our study focused on *S. aureus*, MDA will amplify all DNAs in a complex mixture in a relatively unbiased manner and, thus, should prove equally useful for detection of other pathogens or poly-microbial infections. MDA will not distinguish between DNA from lysed cells versus DNA from intact microorganisms, thus, positive results are not necessarily indicative of bacterial viability, but in the setting of (putatively sterile) synovial fluid any evidence of bacterial DNA may be regarded as abnormal.

The protocol we describe requires only a brief lysis step, obviating the need for any subsequent lengthy nucleic acid purification, and MDA itself demands no specialized equipment or complicated handling. Potentially, the processing required for assay of clinical synovial fluid/aspirates might be minimized even further, although a more expanded and detailed study will be needed to confirm this, as well as to test MDA in a larger number of samples. Although our initial MDA protocol required a 16 hour incubation, a newer commercially available MDA kit (GE Healthcare) requires only 1.5 - 2 hours, possibly bringing even closer the goal of a simple, speedy and reliable PCR-based molecular assay for orthopedic joint infections.

## Conclusion

MDA is a promising technique that may serve to significantly enhance the sensitivity of molecular assays in cases of suspected joint infection while simultaneously reducing the specimen handling required.

## Competing interests

The authors declare that they have no competing interests.

## Authors' contributions

SK, RL, and PS designed the study. SK, LS, and SJ performed the molecular analyses on all samples. SK, RL, LS, JCP and GDE analyzed and interpreted the data. SK, RL, and PS wrote the manuscript. All authors read and approved the final manuscript.
